# Slug overexpression induces stemness and promotes hepatocellular carcinoma cell invasion and metastasis

**DOI:** 10.3892/ol.2014.2037

**Published:** 2014-04-04

**Authors:** YU SUN, GUO-DONG SONG, NING SUN, JIAN-QIU CHEN, SHAO-SHI YANG

**Affiliations:** Department of Surgery, The Second Hospital of Tianjin Medical University, Hexi, Tianjin 300211, P.R. China

**Keywords:** hepatocellular carcinoma, epithelial-mesenchymal transition, Slug, stemness

## Abstract

Detection of metastasis of hepatocellular carcinoma (HCC) is crucial for early diagnosis. Epithelial-mesenchymal transition (EMT) is a common event in the metastasis of tumor cells. Slug and Snail are homologous proteins, which play an important role in EMT. The present study aimed to investigate whether Slug and Snail overexpression is associated with the invasiveness of HCC *in vitro* and *in vivo*. Invasion, colony formation and wound healing assays, as well as flow cytometry analysis, were performed to examine the invasiveness and proliferation capabilities of HepG2 cells following transfection with cNDA or the siRNA of Slug or Snail. The effects of Slug on HCC *in vivo* were examined using a xenograft model. Slug upregulation increased the percentage of cluster of differentiation (CD)133^+^ cells among HepG2 cells, and induced cell invasion and proliferation; whereas Snail upregulation did not affect the cells *in vitro*. The Slug overexpression group exhibited the highest rate of tumor growth compared with the Snail overexpression and control groups *in vivo*. These findings demonstrated that Slug increases the percentage of CD133^+^ cells, promotes the clonigenicity of HCC cells and induces a stronger stemness in Slug-overexpressing cells. These changes activate dormant developmental pathways in invading tumor cells. Thus, Slug may serve as a novel target for HCC prognosis and therapy.

## Introduction

Hepatocellular carcinoma (HCC) is the fifth most common malignancy worldwide and is the third leading cause of cancer-related mortality in China (1). HCC is a rapidly growing tumor associated with a propensity for vascular invasion and metastasis, which results in poor cancer prognoses (2). Considering that methods for early detection of HCC are currently unavailable, the majority of patients present with peritoneal dissemination and distant metastasis at the time of diagnosis (3). Thus, treatment is often unsuccessful and overall survival time is short. Consequently, identifying invasion-related molecules associated with the early and rapid spread of HCC is a focus of investigation.

Invasion and metastasis are the biological hallmarks of malignancy (4). The molecular mechanisms underlying invasion and metastasis are an important area of investigation. Epithelial-mesenchymal transition (EMT) is a common event in the plasticity of tumor cells (5,6). As invasion proceeds, epithelial cell layers lose polarity and cell-cell interaction, which ultimately results in the complex remodeling of the cytoskeleton. EMT is an embryonic trait by which cells adopt a phenotype that is more suitable to migration and invasion (7). A number of molecules associated with tumor invasion and EMT in HCC have been reported, including Twist, Snail and Slug (8–10). However, the molecular changes associated with the metastatic ability in HCC progression have not been clearly determined.

## Materials and methods

### Cell culture and transfection

The HepG2 cell line was obtained from the American Type Culture Collection (Rockville, MD, USA). The cells were cultured in Dulbecco’s modified Eagle’s medium (DMEM) supplemented with 10% fetal bovine serum (FBS; Invitrogen Life Technologies, Carlsbad, CA, USA). pcDNA3.1/Slug, pcDNA3.1/Snail and pcDNA3.1 empty vector (Clontech, Palo Alto, CA, USA) were transfected into the cells using polyethylenimine (cat no. 23966; Polysciences, Inc., Warrington, PA, USA).

### Expression plasmids

Full-length Snail and Slug cDNA were generated using normal human embryo total cDNA, cleaved with *Xho*I/*Eco*RI and subcloned into pcDNA3.1 vectors. The resulting constructs were confirmed by DNA sequencing. The pcDNA3.1 empty vector was used as a cDNA control. Small interfering (si)RNA-coding oligonucleotides against human Snail and Slug were designed and verified for specificity for Snail and Slug. The Snail siRNA targeting sequence was AAGCTGAGCAAGATTCAGACC and the Slug siRNA targeting sequence was CAGGACCTCGCCGCTGCAGAC (siB-cell lymphoma 2 nucleotides 200–221) (11). The U6 promoter with a Snail or Slug siRNA insert was subcloned into pRNA-U6-Neo. A non-silencing siRNA sequence (target sequence, AATTCTCCGAACGTGTCACGT) was used as the negative control.

### Invasion and wound healing assays

The cell migration assay was performed using Transwell cell culture inserts (Invitrogen Life Technologies). The transfected cells were maintained for 48 h and allowed to migrate for an additional 24 h. The passaged cells were stained with crystal violet solution and absorbance was measured at 595 nm. In the wound healing assays, cell motility was assessed by measuring the movement of cells towards the scratch. The speed of wound closure was monitored after 12 and 24 h by measuring the ratio of the distance of the wound at 0 h. Each experiment was performed in triplicate.

### Colony formation assay

The control and transfected cells were seeded into six-well plates at a density of 1,000 cells/well. After two weeks, the clones were fixed in methanol and stained with 2% Giemsa solution (Merck KGaA, Darmstadt, Germany) for 10 min.

### Flow cytometry analysis

Following treatment, the cells were fixed in 75% ethanol and indirectly labeled by incubation with the primary anti-SNAI1 rabbit polyclonal (ab82846; Abcam, Cambridge, MA, USA), anti-SNAI2 mouse monoclonal (ab51772; Abcam), anti-E-cadherin rabbit polyclonal (sc-7870; Santa Cruz Biotechnology, Inc., Santa Cruz, CA, USA), anti-vimentin mouse monoclonal (ab20346; Abcam) and anti-CD133 mouse monoclonal (130080801; Miltenyi Biotech, San Diego, CA, USA) and the secondary antibodies; goat anti-mouse polyclonal IgG horse radish peroxidase (HRP) conjugated antibody (sc-2005, Santa Cruz Biotechnology, Inc.) and goat anti-rabbit polyclonal IgG-HRP conjugated antibody (sc-2004, Santa Cruz Biotechnology, Inc.). The percentage of CD133-positive cells was identified by using flow cytometric analyses with the CD133 monocolonal antibody directly conjugated with phycoerythrin (Miltenyi Biotec, Auburn, CA, USA). All cells were stained and incubated with a CD133-phycoerythrin antibody. A C6 flow cytometer (BD Accuri™, Franklin Lakes, NJ, USA) was used to determine the percent of CD133-positive cells.

### Western blot analysis

Whole cell lysates were resolved by sodium dodecyl sulfate-polyacrylamide gel electrophoresis (Invitrogen Life Technologies) and transferred onto polyvinylidene difluoride membranes (Millipore, Billerica, MA, USA). Blots were blocked and incubated with the mouse monoclonal antibody (anti-SNAI2; ab51772; Abcam) followed by incubation with a secondary antibody (1:2,000; Santa Cruz Biotechnology, Inc.). Blots were developed using an enhanced chemiluminescence detection kit (Amersham Pharmacia Biotech, Inc., Piscataway, NJ, USA). Monoclonal β-actin antibody (1:200; Santa Cruz Biotechnology, Inc.) was used for protein loading analyses.

### Murine xenograft model

Female BALB/c-null mice (age, six weeks) were obtained from the National Institutes of Health (Bethesda, MD, USA) and housed in the animal facilities at the Tianjin Medical University (Tianjin, China), as approved by the Institutional Animal Care and Use Committee. The HepG2 cells (10^7^ cells/ml) were mixed with Matrigel (BD Biosciences, Franklin Lakes, NJ, USA) and subcutaneously injected into the backs of the nude mice (0.1 ml/mouse). The mice were monitored and tumor sizes were measured daily using a caliper for 25 days. The experiments were terminated after 25 days due to the tendency of HepG2 cells to become necrotic and form skin ulcers. The mice were sacrificed by CO_2_ asphyxiation following observations. The tumors were harvested and stored at −80°C for subsequent tests.

### Sulfrodamine B (SRB) cell proliferation assay

Cells were seeded in a 96-well plate (Corning Inc., Corning, NY, USA) at a final concentration of 5000 cells per well follwoing transfection and maintained in DMEM (Gibco-Brl, Grand Island, NY, USA) containing 10% FBS (Gibco-Brl) at 37°C in a humidified atmosphere containing 5% CO_2_. Cell proliferation was analyzed every 8 hours. Initially the cells were washed with distilled water after being fixed with trichloroacetic acid, and were subsequently stained for 10 min with sulforhodamine B (Sigma-Aldrich, St. Louis, MO, USA) dissolved in 1.0% acetic acid (Sigma-Aldrich). The plates were washed with 1.0% acetic acid and allowed to air dry. Finally, 150 μl of 10 mM Tris base (Sigma-Aldrich) was added to each well in order to solubilize the dye, and the absorbance at 490 nm was determined with the use of a microplate reader (Synergy H4, Bio-Tek, Winooski, VT, USA).

### Statistical analysis

All the data were evaluated using SPSS software, version 11.5 (SPSS, Inc., Chicago, IL, USA) and analyzed using the Student’s t-test and analysis of variance. P<0.05 was considered to indicate a statistically significant difference. The significant groups are marked with asterisks as shown in the figures.

## Results

### Slug upregulation increases HepG2 cell proliferation in vitro

**Figure 1:**
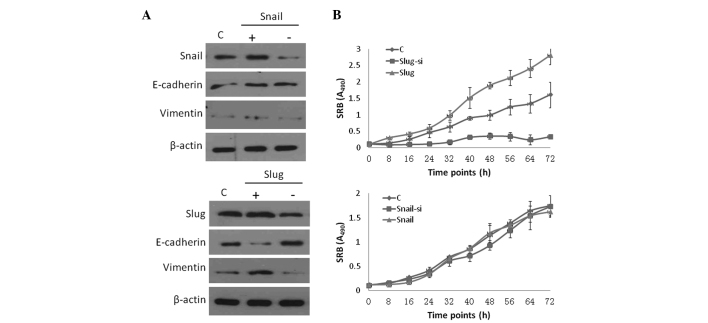
Slug expression promotes HCC cell proliferation. The HepG2 cells were transfected with Slug and Snail expression plasmids and small interfering RNA plasmids. (A) Western blot analysis and (B) the SRB protein assay analyzed the correlation of invasion-related protein levels with HepG2 cell proliferation. HCC, hepatocellular carcinoma; C, control; SRB, sulforhodamine B; si, small interfering (gene knockdown).

The effects of Slug expression on cell invasion, migration and clone formation were investigated. E-cadherin expression is frequently associated with metastatic ovarian carcinoma (12). Metastasis is associated with cell migration and invasion, and the underlying mechanisms are similar to those of EMT. The invasiveness and migration capability of HepG2 cells in the Slug and Snail transfection and knockdown groups were examined. Western blot analysis revealed a significant difference in the ectopic transfection groups compared with the control group. Slug and Snail were upregulated in the overexpressed group and were downregulated in the siRNA group (Fig. 1A). The sulforhodamine B protein assay was used to measure cell numbers and a significant difference in cell proliferation was observed between the Slug overexpression and knockdown groups. The cells exhibited a significant decrease in cell proliferation in the Slug knockdown group, whereas cell proliferation was significantly increased in the overexpressed group compared with the control group (P<0.01). Snail expression did not significantly affect cell proliferation (P>0.01).

### Slug upregulation increases HCC cell invasion, migration and clone formation in vitro

**Figure 2:**
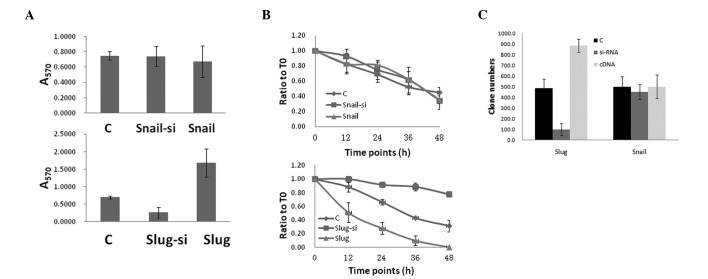
Slug expression promotes HepG2 cell invasion, migration and clone formation. The HepG2 cells were transfected with Slug and Snail expression plasmids and si RNA plasmids. (A) Invasion and (B) wound healing assays tested the migration capability and invasiveness of HepG2 cells. (C) A colony formation assay analyzed the effects of Slug and Snail on the clone formation of HepG2 cells. C, control; siRNA, small interfering RNA.

The HepG2 cell cultures were analyzed for functional changes in migration, invasion and clone formation following transfection with Slug and Snail, separately. Compared with the transfection and control groups, the Slug overexpression group exhibited a significantly increased activity in the migration, invasion and clonigenicity assays (Fig. 2). The effects of non-uniform transfection efficiency were minimized by selecting Slug siRNA-transfected cells for slug knockdown, and the Slug knockdown group revealed decreased activity in the migration, invasion and clonigenicity assays compared with the control group. Notably, Snail, which shares structural homology with Slug, did not promote cell migration.

### Slug upregulation increases the percentage of cluster of differentiation (CD)133^+^ cells in HCC cells

**Figure 3:**
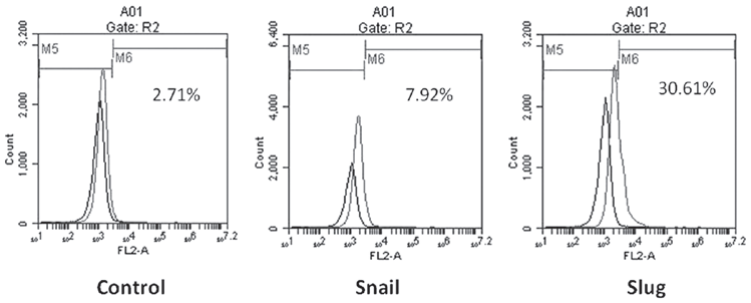
Slug expression increases the number of CD133^+^ cells among the HepG2 cells. Flow cytometry analyzed the percentage of CD133^+^ cells among HepG2 cells transfected with Slug and Snail cDNA, and in the controls. CD, cluster of differentiation.

Stem cells play an important role in tumor cell plasticity and proliferation during tumor metastasis, and are strongly associated with EMT. In this study, flow cytometry examined the percentage of CD133^+^ cells in the Slug overexpression and knockdown groups. The CD133^+^ cells accounted for 47.5% of the cells in the Slug overexpression group, 8.7% in the control transfection group and 0.4% in the Slug knockdown group (Fig. 3). No significant difference in the percentage of CD133^+^ cells was observed between the Snail overexpression, Snail knockdown and control groups. These findings suggest that synergism between Slug and CD133^+^ cells increases cell proliferation, migration and invasion.

### Coexpression of CD133 and Slug correlates with tumor proliferation

**Figure 4:**
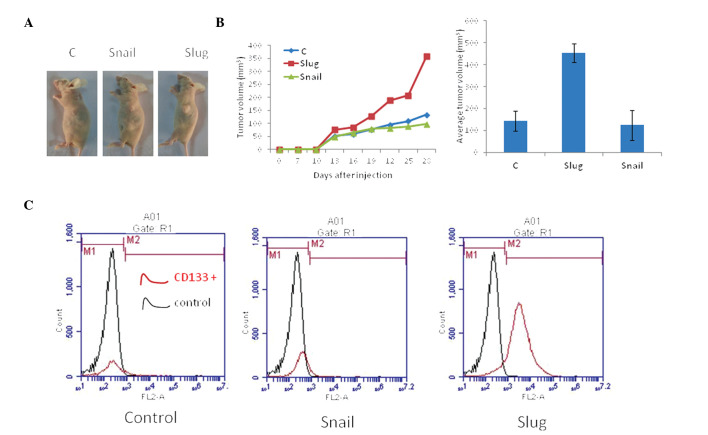
Slug expression increases tumor growth and the percentage of CD133^+^ cells *in vivo*. The HepG2 cells transfected with Slug and Snail cDNA were injected into nude mice. (A and B) The tumor volume was examined and recorded, and (C) the percentage of CD133^+^ cells was determined by flow cytometry. C, control; CD, cluster of differentiation.

A murine xenograft model was used to examine the *in vivo* effects of Slug and Snail expression on tumor development. The HepG2 cells were utilized to establish xenografts in nude mice. Nodule formation and growth (volume) were monitored over 25 days. The Slug overexpression group displayed the highest rate of tumor growth compared with the Snail overexpression (60%) and control (50%) groups (Fig. 4). The cells were collected from the tumor tissues and analyzed using flow cytometry. The findings demonstrated a significant increase in CD133^+^ cells in the Slug overexpression group compared with the control group, but no significant difference was observed between the Snail overexpression and the control groups (Fig. 4).

## Discussion

HCC invasiveness is a key step that results in metastasis and a poor prognosis (1,11); therefore, the underlying molecular mechanisms are a focus of investigation. EMT plays an important role in the development of tissues during embryogenesis (13). However, similar cell changes are recapitulated during pathological processes, such as in cancer progression. Previous studies on EMT have focused on cancer cell invasion and metastasis (6,14). Several developmental genes that induce EMT act as E-cadherin repressors. The first of these genes is the Snail family of zinc-finger protein transcription factors, which is a DNA-binding factor family that recognizes E-box motifs in target promoters, such as E-cadherin. Slug is homologous to Snail and was the first transcriptional repressor of E-cadherin to be described along with other non-Snail transcriptional repressors of E-cadherin [such as, E47, delta-crystallin/E2-box factor 1/zinc finger E-Box-binding homeobox (Zeb) 1 and Smad-interacting protein 1/Zeb2] (15–18). These repressors are tightly regulated at the transcriptional level and/or by subcellular localization. Insights into the underlying mechanisms of EMT regulation may provide novel chemotherapeutic and antifibrotic therapies.

Tumor progression and invasion are complex biological processes that involve the remodeling of stromal tissue by invading cells (12,19–21). Slug and Snail are members of an evolutionarily conserved family of zinc-finger transcription factors. Slug and Snail are expressed in the intermediate mesoderm and the metanephric mesenchyme during renal development, and are downregulated prior to epithelial differentiation (22,23). The kidneys developed normally in mice with a loss-of-function mutation in Snail, which suggests the functional redundancy of Snail and Slug (24,25). However, the E-cadherin repressors, Snail, Slug and E47, produce different genetic profiles when overexpressed in ovarian tumor cells, suggesting differential regulation of these transcription factors (8,26–28). In the present study, the constitutive expression of Slug increased invasion by inducing EMT and the results obtained following gene knockdown were consistent with those of a previous study (21). Our findings suggest that Slug induces EMT, increases the percentage of CD133^+^ cells, promotes the clonigenicity of HCC cells and induces a stronger stemness. These changes activate dormant developmental pathways in invading tumor cells; therefore, suppressing invasion-related molecules, such as Slug, may present an important mechanism to suppress metastasis. Furthermore, increased Snail levels did not significantly affect HCC cell proliferation, migration, invasion, clonigenicity or the number of CD133^+^ cells. Thus, Snail proteins may have a polarizing effect on HCC tissue growth.

In conclusion, our findings demonstrated that Slug upregulation increased the number of CD133^+^ cells, which is important for EMT and proliferation of ovarian cancer cells. Therefore, Slug may be a potential new target for preventing tumor invasion and metastasis.

## References

[1] Yang MH, Chen CL, Chau GY (2009). Comprehensive analysis of the independent effect of twist and snail in promoting metastasis of hepatocellular carcinoma. Hepatology.

[2] Hanazaki K, Matsushita A, Nakagawa K, Misawa R, Amano J (2005). Risk factors of long-term survival and recurrence after curative resection of hepatocellular carcinoma. Hepatogastroenterology.

[3] Yeoman AD, Al-Chalabi T, Karani JB (2008). Evaluation of risk factors in the development of hepatocellular carcinoma in autoimmune hepatitis: Implications for follow-up and screening. Hepatology.

[4] Hanazaki K, Matsushita A, Nakagawa K, Misawa R, Amano J (2005). Risk factors of intrahepatic recurrence after curative resection of hepatocellular carcinoma. Hepatogastroenterology.

[5] Savagner P (2010). The epithelial-mesenchymal transition (EMT) phenomenon. Ann Oncol.

[6] Bacac M, Stamenkovic I (2008). Metastatic cancer cell. Annu Rev Pathol.

[7] Kang Y, Massagué J (2004). Epithelial-mesenchymal transitions: twist in development and metastasis. Cell.

[8] Smit MA, Geiger TR, Song JY, Gitelman I, Peeper DS (2009). A Twist-Snail axis critical for TrkB-induced epithelial-mesenchymal transition-like transformation, anoikis resistance, and metastasis. Mol Cell Biol.

[9] Hotz B, Arndt M, Dullat S, Bhargava S, Buhr HJ, Hotz HG (2007). Epithelial to mesenchymal transition: expression of the regulators snail, slug, and twist in pancreatic cancer. Clin Cancer Res.

[10] Šošić D, Richardson JA, Yu K, Ornitz DM, Olson EN (2003). Twist regulates cytokine gene expression through a negative feedback loop that represses NF-kappaB activity. Cell.

[11] McCawley LJ, Matrisian LM (2001). Tumor progression: defining the soil round the tumor seed. Curr Biol.

[12] Sun T, Zhao N, Zhao XL (2010). Expression and functional significance of Twist1 in hepatocellular carcinoma: its role in vasculogenic mimicry. Hepatology.

[13] Zhou C, Liu J, Tang Y, Liang X (2012). Inflammation linking EMT and cancer stem cells. Oral Oncol.

[14] Childs G, Segall JE (2012). Twists and turns of invasion. Nat Cell Biol.

[15] Firulli AB, Conway SJ (2008). Phosphoregulation of Twist1 provides a mechanism of cell fate control. Curr Med Chem.

[16] Yang MH, Wu KJ (2008). TWIST activation by hypoxia inducible factor-1 (HIF-1): implications in metastasis and development. Cell Cycle.

[17] Martin A, Cano A (2010). Tumorigenesis: Twist1 links EMT to self-renewal. Nat Cell Biol.

[18] Pietras K, Ostman A (2010). Hallmarks of cancer: interactions with the tumor stroma. Exp Cell Res.

[19] Peng JY, Wang Y (2010). Tumor stroma: A determinant role in local recurrence of rectal cancer patients receiving total mesorectal excision?. Med Hypotheses.

[20] Erenpreisa J, Cragg MS (2007). Cancer: a matter of life cycle?. Cell Biol Int.

[21] Sun T, Sun BC, Zhao XL (2011). Promotion of tumor cell metastasis and vasculogenic mimicry by way of transcription coactivation by Bcl-2 and Twist1: a study of hepatocellular carcinoma. Hepatology.

[22] Timmerman LA, Grego-Bessa J, Raya A (2004). Notch promotes epithelial-mesenchymal transition during cardiac development and oncogenic transformation. Genes Dev.

[23] Alonso-Magdalena P, Brössner C, Reiner A (2009). A role for epithelial-mesenchymal transition in the etiology of benign prostatic hyperplasia. Proc Natl Acad Sci USA.

[24] Ikenouchi J, Matsuda M, Furuse M, Tsukita S (2003). Regulation of tight junctions during the epithelium-mesenchyme transition: direct repression of the gene expression of claudins/occludin by Snail. J Cell Sci.

[25] Carver EA, Jiang R, Lan Y, Oram KF, Gridley T (2001). The mouse snail gene encodes a key regulator of the epithelial-mesenchymal transition. Mol Cell Biol.

[26] Smith JP, Pozzi A, Dhawan P, Singh AB, Harris RC (2009). Soluble HB-EGF induces epithelial-to-mesenchymal transition in inner medullary collecting duct cells by upregulating Snail-2. Am J Physiol Renal Physiol.

[27] Usami Y, Satake S, Nakayama F (2008). Snail-associated epithelial-mesenchymal transition promotes oesophageal squamous cell carcinoma motility and progression. J Pathol.

[28] Medici D, Hay ED, Olsen BR (2008). Snail and Slug promote epithelial-mesenchymal transition through beta-catenin-T-cell factor-4-dependent expression of transforming growth factor-beta3. Mol Biol Cell.

